# Real-World Results of Aflibercept versus Ranibizumab for the Treatment of Exudative AMD Using a Fixed Regimen

**DOI:** 10.1155/2018/9276580

**Published:** 2018-06-06

**Authors:** Joana Providência, Tiago M. Rodrigues, Mariana Oliveira, João Bernardes, João Pedro Marques, Joaquim Murta, Rufino Silva

**Affiliations:** ^1^Department of Ophthalmology, Centro Hospitalar e Universitário de Coimbra, Coimbra, Portugal; ^2^Instituto de Medicina Molecular, Faculdade de Medicina, Universidade de Lisboa, Lisboa, Portugal; ^3^Faculty of Medicine, University of Coimbra, Coimbra, Portugal; ^4^Association for Innovation and Biomedical Research on Light (AIBILI), Coimbra, Portugal

## Abstract

Intravitreal injections of antivascular endothelial growth factors have been considered a milestone in the treatment of neovascular age-related macular degeneration (nAMD). However, the increasing incidence of AMD and the burden of visits and injections overcharge both the patient and the healthcare systems. Real-world solutions depend on treatment protocols aimed at optimizing the number of clinical visits while guaranteeing good functional outcomes. We performed a retrospective analysis of 72 eyes from 63* naïve* patients diagnosed with nAMD that underwent a fixed intravitreal protocol consisting of bimonthly injections after a three-month loading dose, with either Aflibercept or Ranibizumab (no predefined criteria for treatment selection). Best corrected visual acuity (BCVA) and optical coherence tomography were analyzed at baseline and during follow-up clinical visits (months 3, 6, 12, and 18). From the included participants, 42 followed a fixed regimen with Aflibercept and 30 with Ranibizumab. At the 12-month visit, there was not a statistically significant difference in the mean change of BCVA between the two groups (p=0.121); however, the mean difference in the central retinal thickness was significantly superior in the Aflibercept group (-142.2 versus -51.5, p=0.011). The described fixed regimen seems to be efficient in the treatment of nAMD in a clinical practice setting.

## 1. Introduction

Age-related macular degeneration (AMD) is the leading cause of irreversible vision loss in the elderly in developed countries, with an estimated prevalence in the Portuguese population of 12,48% and 1,16% for the early and late forms, respectively [1, 2].

Neovascular AMD (nAMD), an advanced stage of the disease, is characterized by the growth and leakage of new blood vessels arising from the choroid, with vascular endothelial growth factor (VEGF) playing a key role in macular scarring and, consequently, loss of central vision. Intravitreal anti-VEGFs are one of the milestones of nAMD treatment, being used for more than ten years now [3]. The efficacy and safety of Ranibizumab, administered monthly, was established in the MARINA [4] and ANCHOR [5] phase III trials. More recently, the noninferiority of Aflibercept administered bimonthly after a loading dose of 3 monthly injections was described in the VIEW 1 and 2 trials, the first prospective randomized controlled trials comparing Aflibercept to Ranibizumab [6].

Although the best functional outcomes in clinical trials were achieved using monthly injections, alternative regimens were shown to produce reasonable outcomes with significantly less visits and injections [7]. In the PRONTO study [8] and in the phase 3 clinical trial HARBOR [9], patients treated in* a pro re nata* (PRN, as needed) regimen based on optical coherence tomography (OCT) and VA criteria achieved comparable VA gains at month 24, as the fixed monthly Ranibizumab arm of the studies, with fewer number of injections. Another regimen that proved safe and effective is the treat-and-extend (T&E) regimen [10–12]. In this regimen, patients are treated at each visit and the interval between visits (and treatments) is gradually extended or shortened, depending on the absence or presence of fluid on OCT, respectively. Finally, fixed regimens provide also an alternative to the monthly schemes. However, in two phase 3 studies (PIER and EXCITE) there was a decline in VA when a quarterly dosing scheme of Ranibizumab was introduced, compared with the monthly administration [13, 14].

The rising number of affected individuals along with the need to perform monthly injections and clinical evaluations overcharges both the healthcare systems and the patients, making utopic the implementation of clinical trial-like treatment protocols. However, there is lack of knowledge regarding the real-world efficacy of intravitreal anti-VEGFs. The published data suggests that patients are usually undertreated in a clinical practice setting. A recently published paper highlights the similarity of real-world outcomes in patients treated with a similar number of injections of Ranibizumab or Aflibercept, despite the differences in the approved treatment regimens [15]. New strategies need to be defined to minimize the number of hospital visits, in order to treat with success the greatest number of patients, considering the clinical resources available.

The aim of this study was to compare the real-world efficacy of intravitreal Aflibercept and Ranibizumab administrated in a fixed regimen, for the treatment of nAMD in a tertiary ophthalmology department in Portugal.

## 2. Methods

### 2.1. Study Design

This is a single-center, retrospective, comparative, nonrandomized study.

### 2.2. Study Population

Treatment-naïve patients starting a fixed treatment protocol for nAMD between January 2015 and April 2016 and with a minimum follow-up of 18 months were included. The diagnosis and staging of AMD were performed by experienced retina specialists. The physician in charge of the patient's treatment was responsible for the decision of starting the protocol with either Aflibercept or Ranibizumab and this was not based on any set of predefined criteria such as VA and CNV type.

Exclusion criteria included the diagnosis of any other vitreoretinal diseases, significant media opacities that precluded the observation of the ocular fundus, refractive errors greater than 6 diopters of spherical equivalent, past history of retinal surgery, and diagnosis of diabetes mellitus with or without diabetic retinopathy. Patients with major delays in the initiation of the treatment protocol, defined as more than 4 months without intravitreal injection of anti-VEGF, were also excluded.

### 2.3. Study Protocol

Before enrolment to the treatment protocol, all participants underwent a complete ophthalmological examination, including best corrected visual acuity (BCVA), intraocular pressure measurement, slit-lamp biomicroscopy, and detailed fundus exam. In the same visit, all subjects were imaged with OCT (Cirrus, Zeiss), fluorescein angiography, and indocyanine-green angiography, which were used to perform the diagnosis and classification of nAMD, according to Age-Related Eye Disease Study Classification [16].

**Figure 1:**
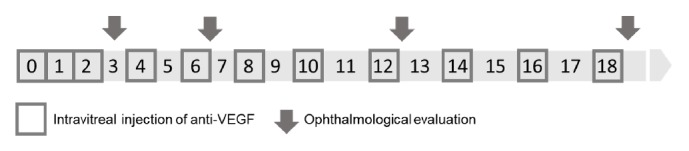
Schematic representation of intravitreal injections protocol for the treatment of nAMD. nAMD: neovascular age-related macular degeneration.

The treatment protocol consisted of a loading dose of 3 monthly intravitreal injections of anti-VEGF (months 0, 1, and 2) followed by a complete ophthalmologic evaluation (month 3), from which OCT and BCVA, recorded as ETDRS letter score, were documented. After this clinical visit, the patients underwent a fixed regimen of three bimonthly injections (months 4, 6, 8, 10, 12, 14, 16, and 18), separated by complete ophthalmologic evaluations (months 6, 12, and 18), from which OCT and BCVA data were extracted (Figure 1).

We analyzed the data from OCT and BCVA at the baseline, after the loading dose of intravitreal anti-VEGF (3rd month) and at 6, 12, and 18 months. The central retinal thickness (CRT) was obtained from automated maps according to the conventional Early Treatment Diabetic Retinopathy Study (ETDRS) grid. The OCT scans were graded for the presence of intraretinal fluid, subretinal fluid, vitreoretinal interface changes, and presence of disciform scars or geographic atrophy.

### 2.4. Outcome Measures

The main endpoint was the mean change from baseline in BCVA and in central retinal thickness at the 12-month visit. Secondary endpoints included the mean change from baseline in BCVA at the 3-, 6-, and 18-month visits. Furthermore, we analyzed the proportion of patients gaining or losing more than 15 ETDRS letters in each group.

Regarding the OCT analysis, we recorded the mean change in central retinal thickness at the 3-, 6-, and 18-month visits. All OCT scans were graded for the presence of subretinal and intraretinal fluid and for the presence of changes of the vitreoretinal interface, including vitreomacular adhesion, traction, or presence of macular hole. The proportion of patients achieving a “dry macula”, defined as the absence of both intra- and subretinal fluid, was compared between the two groups.

Safety endpoints included any reported adverse event that could be related to the treatment.

### 2.5. Statistical Analysis

The study population demographics were analyzed with traditional descriptive measures, with means and standard deviations for continuous variables and percentages for categorical variables. The eye was defined as the unit of analysis. In order to ascertain the existence of imbalances between the treatment groups, P values using unpaired* t*-test for continuous independent variables were reported. For dichotomic variables, a Chi-square test was performed.

We defined two primary outcomes: mean change from the baseline in BCVA and central retinal thickness at the 12-month visit. For the analysis of the primary outcome measures, we used multilevel mixed effect linear models in order to include both eyes of some participants (random factor), whenever possible. Multilevel mixed models take into account the fact that the eyes of the same patient are correlated. The relationship between covariates and the primary outcomes was first evaluated in univariate models; subsequently, covariates were included in the multivariate model, in order to control for confounding. We also evaluated change from baseline in BCVA and central retinal thickness at the 18-month visit as secondary outcome measures. A similar strategy was used to build the models to analyze these outcomes.

All statistics were performed on STATA (version 14.2, StataCorp LLC, College Station, TX, USA). Graphical representations were built on SPSS (IBM SPSS Statistics 23). A p value of < 0.05 was considered statistically significant.

## 3. Results

### 3.1. Study Population

We included 72 treatment-naïve nAMD eyes from 63 patients initiating a fixed treatment protocol with either Aflibercept or Ranibizumab between January 2015 and April 2016. Of the included eyes, 42 (58,33%) started treatment with Aflibercept and 30 (41,67%) with Ranibizumab (**Table 1**).

Similar baseline demographic characteristics were observed in both arms of the study. However, patients in the Aflibercept group were significantly younger (mean age 78.02 ± 7.17 versus 82.15 ± 6.12 in the Ranibizumab group, p=0.016). The baseline clinical characteristics including best corrected visual acuity and mean central retinal thickness were similar in both groups. The mean number of injections both at 12 and 18 months was slightly higher in the Aflibercept group (5.86 and 7.57, respectively, versus 5.47 and 6.59 in the Ranibizumab group), although the difference was not statistically significant.

Three eyes in the Ranibizumab group, versus none in the Aflibercept group, discontinued the treatment during the follow-up period due to advanced disciform scars and poor functional prognosis.

### 3.2. Best Corrected Visual Acuity and Central Retinal Thickness at 12-Month Visit

**Figure 2:**
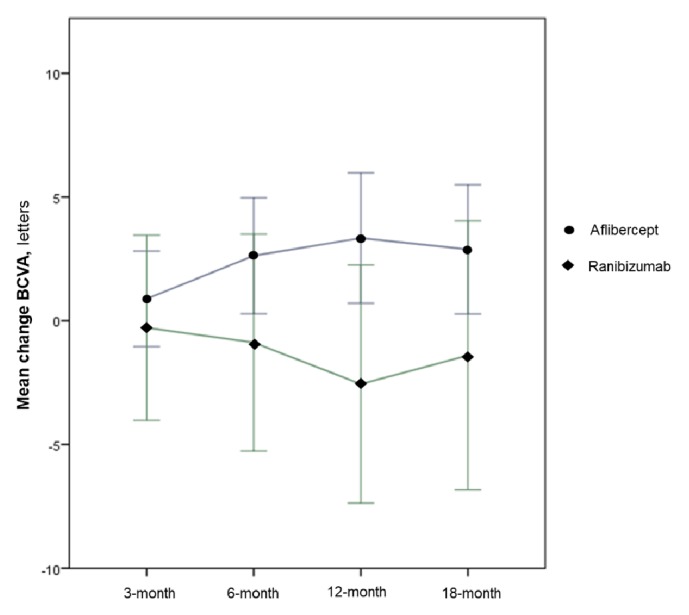
Mean difference in BCVA in both treatment arms during the follow-up. BCVA: best corrected visual acuity.

At month 12, the mean (± SD) change from baseline in BCVA was +2,74 (±7,06) letters for the Aflibercept group and -3,07 (±11,06) letters for the Ranibizumab group (**Figure 2**). This difference was not statistically significant (p=0.121) after controlling for confounding factors, namely, age, number of injections, visual acuity, and central retinal thickness at baseline (**Table 2**).

We observed a mean difference in the central retinal thickness of -142.0 (-470 to +57) versus -51.52 (-407 to +260) in the Aflibercept and Ranibizumab groups (p=0.011), respectively, which was significant after controlling for the confounding factors age, number of injections, visual acuity, and visual acuity at baseline.

### 3.3. Best Corrected Visual Acuity at Other Visits

At the first visit following the 3 monthly injections, the mean (± SD) change in BCVA from baseline was +1,05 (±5,64) for the Aflibercept group and -1,13 (±8,9) for the Ranibizumab group, with a mean BCVA of 54,37 (±19,1) letters for the Aflibercept group versus 51,38 (±20,5) letters for the Ranibizumab group.

At the 6-month visit, the mean change from baseline continued to improve in the Aflibercept group, with +1,83 (±10,88) letters, but slightly declined in the Ranibizumab group, with -2,63 (±12,46) letters. Finally, at month 18, the mean (± SD) change from baseline in BCVA was +2,05 (± 8,37) letters for the Aflibercept group and -1,4 (±13,17) letters for the Ranibizumab group. This difference was not statistically significant (p=0.581) after controlling for confounding factors, namely, age, number of injections, visual acuity, and central retinal thickness at baseline (**Table 2**).

At the 18-month visit, 7,1% of the eyes in the Aflibercept group registered a loss of more than 15 ETDRS letters, against 16,7% eyes on the Ranibizumab group. In the same visit, the proportion of patients gaining more than 15 ETDRS letters was also equivalent between the two groups (7,1% versus 3,3%, respectively).

### 3.4. Mean Central Retinal Thickness

**Figure 3:**
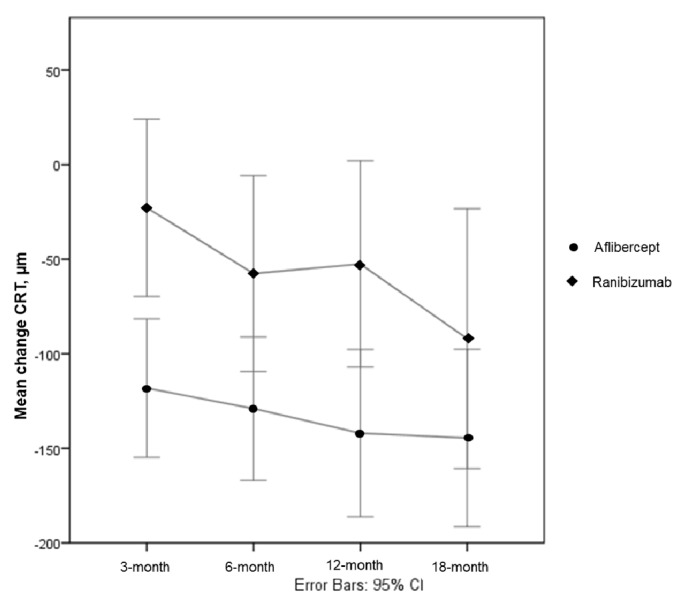
Mean difference in CRT in both treatment arms during the follow-up. CRT: central retinal thickness.

The mean difference in the CRT was superior in the Aflibercept group in all visits (**Table 2**). After the loading dose, we observed a mean difference of -119,4 (-366 to +7) versus -28.2 (-347 to +220) in the Aflibercept and Ranibizumab groups, respectively. After the 18-month visit, a statistically significant difference was maintained, with a mean reduction of 147,2 (-482 to +235) versus 50,0 (-501 to +260) for the Aflibercept and Ranibizumab groups (p=0,041), respectively, even after controlling for the confounding variables, namely, age, number of injections, visual acuity, and central retinal thickness at baseline (Figure 3).

### 3.5. Other OCT Analyses

After the loading dose, 59,5% (n=25) of eyes treated with Aflibercept achieved a dry macula, against 23,3% (n=7) of eyes treated with Ranibizumab. This difference was maintained at the final visit, with 71,4% (n=30) of Aflibercept patients achieving a dry macula, against 40,0% (n=12) of Ranibizumab eyes.

### 3.6. Adverse Events

Intravitreal injections of the studied anti-VEGFs were generally well tolerated and no adverse events were reported on either group, during the 18-month follow-up period.

## 4. Discussion

We present a single-center retrospective analysis of the real-world treatment of nAMD with a fixed regimen of intravitreal injections of either Aflibercept or Ranibizumab. Our results revealed equivalent mean changes in BCVA at 12 months in both groups, despite a not statistically significant difference favoring the Aflibercept group. This not statistically significant difference in the mean change in BCVA was observed during all the clinical visits of the 18-month follow-up period and might reveal a trend to a superior BCVA score in the Aflibercept group that our sample was not empowered to detect. However, both studied regimens were demonstrated to maintain the BCVA during the follow-up period, despite the reduced number of clinical visits and intravitreal injections when compared to previous literature. Losses > 15 letters occurred only on 7,1% and 16,7% of the eyes treated with Aflibercept and Ranibizumab, respectively.

To our knowledge, this is the first analysis comparing a real-world fixed regimen protocol of Aflibercept and Ranibizumab. Despite the differences in their approved treatment regimens, both anti-VEGFs have been used in a similar fashion in clinical practice, with a comparable number of injections being given to nAMD patients undergoing treatment with both drugs. Previous studies have reported a reduced efficacy of Ranibizumab when administered in protocols with less frequent injections. In the PIER study [13], the VA benefit obtained with quarterly dosing was not as robust as the monthly dosing described in ANCHOR and MARINA studies [4]. In the EXCITE study, both monthly and quarterly Ranibizumab treatment regimens maintained BCVA in patients with nAMD, but with superior letter-gains in the monthly regimen [14]. In the same study, the quarterly regimen could not achieve the noninferiority compared to the already approved monthly regimen. Although we used a regimen with more frequent injections (every 2 months instead of every 3 months), we succeeded in maintaining BCVA in a reasonable proportion of treated eyes, even though our results are inferior to the ones reported with monthly regimens. The VIEW 1 and 2 reported that intravitreal Aflibercept dosed monthly or every 2 months after 3 initial monthly doses produced similar efficacy and safety outcomes as monthly Ranibizumab. However the VIEW protocols have important limitations as the treatment regimens are different between the study arms, as Ranibizumab is only administered monthly. In our study, both Aflibercept and Ranibizumab are used in a similar treatment regimen.

The benefits of anti-VEGF treatment with either Aflibercept or Ranibizumab were also reflected in the anatomic measures evaluated with OCT. However, when both treatment groups were compared, a statistically significant difference in the CRT was observed throughout follow-up, favoring the Aflibercept group, even after controlling for the confounding factors. This is in agreement with the possibility of an insufficient dose of Ranibizumab being administered in this fixed regimen.

A current challenge is to determine an optimized regimen that provides best efficacy results without causing a significant burden to the patients and the healthcare system. The 3-month loading dose seems to be of benefit for both Aflibercept and Ranibizumab. However, the percentage of patients with early persistent retinal fluid was superior in the group receiving Ranibizumab. These results are in agreement with the post hoc analysis of the VIEW 1 and 2 trials, where early persistent fluid was shown to respond better to Aflibercept than to Ranibizumab [17]. A recently published meta-analysis [18] states that although Aflibercept and Ranibizumab demonstrated comparable effects for treatment-naïve nAMD in the real world, Aflibercept was significantly more effective in patients with initial reduced visual acuity (defined as logMAR > 0.6 [ < 55 letters]). The same meta-analysis stated that patients treated with Aflibercept in a PRN regimen required fewer injections compared to Ranibizumab-treated patients. Further studies comparing fixed regimens, PRN, and treat-and-extend protocols, including the best time frame for extending visits, need to be conducted in order to optimize treatment strategies.

Our study has several limitations. First of all, a larger patient cohort would possibly have found more significant differences between groups that were not apparent in this study. The reduced number of included subjects reflects the reality of our clinical practice, with an important number of patients having to be excluded from this analysis because of premature discontinuation of the protocol (drop outs, agenda issues, etc.). Secondly, conducting a retrospective study without any preestablished criteria to use either drug, we cannot exclude differences in the physician-related anti-VEGF selection between patients included in each treatment group. Still, the fact that both groups had similar baseline VA scores minimizes this issue. Thirdly, according to our protocol, it would be expected to ideally have 11 intravitreal injections at the 18-month visit. However, agenda related issues are not infrequent and we noted a delay in the protocol in both treatment arms that might be related to our less satisfactory results when compared to previously published literature regarding trials with a superior number of intravitreal injections of anti-VEGF.

In conclusion, despite anatomic results favoring the Aflibercept group, BCVA scores did not significantly differ between the two drugs, both at the 18-month visit and throughout follow-up. A fixed regimen of bimonthly intravitreal Aflibercept or Ranibizumab following a 3-month loading dose seems to be efficient in the treatment of nAMD in a clinical practice setting, with less clinical visits compared to monthly regimens. Larger and prospectively designed studies are needed to understand whether there is a significant difference in treatment responses between the drugs, allowing us to define more accurate treatment protocols that can benefit the largest number of patients.

## Figures and Tables

**Table 1:** Demographic and clinical characteristics of the included eyes.

**Characteristics**	**Aflibercept **	**Ranibizumab**	**Total**	**P- value**
**No of Patients (%)**	39 (61,90)	24 (38,10)	63 (100)	
**No of eyes (%)**	42 (58,33)	30 (41,67)	72 (100)	

**Demographic Characteristics**				

**Age, mean ± SD**	78,02 ± 7,17	82,15 ± 6,12	79,81 ± 7,01	0,016

**Gender, n (%)**				0,117
Female	20 (47,6)	20 (66,7)	40 (55,6)	
Male	22 (52,4)	10 (33,3)	32 (44,4)	

**Clinical Characteristics**				

**Baseline BCVA, ** **mean ± SD**	53,33 ± 17,02	52,16 ± 14,4	52,82 ± 17,12	0,661

**Baseline CRT, ** **mean ± SD**	408,14 ± 117,84	379,71 ± 110,51	396,77 ± 115,01	0,786

**No Injections ** **12m, mean ± SD**	5,86 ± 0,84	5,47 ± 1,14	5,68 ± 0,99	0,055

**No Injections ** **18m, mean ± SD**	7,57 ± 1,48	6,59 ± 1,45	7,15 ± 1,61	0,064

**Protocol interruption, n (%)**	0	3 (10)	3 (4,17)	0,041

No: number, SD: standard deviation, BCVA: best corrected visual acuity, CRT: central retinal thickness, m: month

**Table 2:** Univariate and multivariate analysis using a multilevel linear regression model.

**Primary Analysis: 12 Months**				
***Mean Change in BCVA***				

	Univariate		Multivariate	

	Beta (95% CI)	P-value	Beta (95% CI)	P-value

Treatment Group	-3.39 (-8.04, 1-26)	0.153	-3.86 (-8.75, 1.02)	0.121

Age	0.16 (-0.16, 0.48)	0.324	0.11 (-0.24, 0.45)	0.545

No Inj (12 mo)	1.09 (-1.50, 3.68)	0.408	1.88 (-0.76, 4.52)	0.163

BCVA baseline	-0.04 (-0.17, 0.09)	0.577	-0.10 (-0.23, 0.04)	0.169

CRT baseline	-0.02 (-0.04, -0.001)	0.037	-0.03 (-0.04, -0.01)	0.010

***Mean Change in CRT***				

	Univariate		Multivariate	

	Beta (95% CI)	P-value	Beta (95% CI)	P-value

Treatment Group	49.20 (-7.56, 105.95)	0.009	24.44 (-5.86, 54.47)	0.011

Age	3.51 (-0.30, 7.32)	0.071	0.73 (-1.49, 2.98)	0.493

No Inj (12 mo)	9.28 (22.55, 41.10)	0.568	9.14 (-7.15, 25.5)	0.270

BCVA baseline	2.76 (-0.17, 0.09)	<0.001	0.46 (-0.21, 2.01)	0.016

CRT baseline	-0.80 (-0.92, -0.67)	<0.001	-0.72 (-0.86, -0.59)	<0.001

**Secondary Analysis: 18 months**				

***Mean change in BCVA***				

	Univariate		Multivariate	

	Beta (95% CI)	P-value	Beta (95% CI)	P-value

Treatment Group	-2.86 (-7.72, 2.00)	0.249	-1.42 (-6.44, 3.61)	0.581

Age	-0.02 (-0.36, 0.32)	0.925	-0.06 (-0.41, 0.29)	0.743

No Inj (18 mo)	0.50 (-1.17, 2.16)	0.561	1.01 (-0.72, 2.74)	0.254

BCVA baseline	-0.02 (-0.16, 0.12)	0.764	-0.08 (-0.22, 0.07)	0.309

CRT baseline	-0.01 (-0.03, -0.01)	0.158	-0.02 (-0.03, 0.001)	0.073

***Mean change in CRT***				

	Univariate		Multivariate	

	Beta (95% CI)	P-value	Beta (95% CI)	P-value

Treatment Group	64.09 (7.08, 121.09)	0.028	39.85 (1.66, 78.04)	0.041

Age	3.44 (-0.50, 7.49)	0.087	-0.43 (-3.13, 2.28)	0.756

No Inj (18 mo)	3.00 (-17.38, 23.37)	0.773	6.55 (-6.67, 19.78)	0.332

BCVA baseline	1.72 (0.08, 3.35)	0.040	-0.16 (-1.30, 0.97)	0.778

CRT baseline	-0.81 (-0.95, -0.66)	<0.001	-0.79 (-0.96, -0.63)	<0.001

BCVA: best corrected visual acuity, No Inj: number of injections, CRT: central retinal thickness, mo: months

## Data Availability

The data used to support the findings of this study are available from the corresponding author upon request.
